# Variation in assessment and standard setting practices across UK undergraduate medicine and the need for a benchmark

**DOI:** 10.5116/ijme.560e.c964

**Published:** 2015-10-31

**Authors:** Margaret MacDougall

**Affiliations:** Centre for Population Health Sciences, College of Medicine and Veterinary Medicine, University of Edinburgh, UK

**Keywords:** assessment, benchmarking, national licensing exam, standard setting, undergraduate medical education

## Abstract

**Objectives:**

The principal aim of this study is to provide an account of variation in UK undergraduate medical assessment styles and corresponding standard setting approaches with a view to highlighting the importance of a UK national licensing exam in recognizing a common standard.

**Methods:**

Using a secure online survey system, response data were collected during the period 13 - 30 January 2014 from selected specialists in medical education assessment, who served as representatives for their respective medical schools.

**Results:**

Assessment styles and corresponding choices of standard setting methods vary markedly across UK medical schools. While there is considerable consensus on the application of compensatory approaches, individual schools display their own nuances through use of hybrid assessment and standard setting styles, uptake of less popular standard setting techniques and divided views on norm referencing.

**Conclusions:**

The extent of variation in assessment and standard setting practices across UK medical schools validates the concern that there is a lack of evidence that UK medical students achieve a common standard on graduation. A national licensing exam is therefore a viable option for benchmarking the performance of all UK undergraduate medical students.

## Introduction

This survey-based study aims to provide an up-to-date investigation of the variety of assessment instruments and corresponding standard setting approaches as used in UK medical schools. The interpretation of the term *standard setting* is akin to that used by Bejar in referring to “the methodology used to define *levels* of achievement or proficiency and the *cutscores* corresponding to those *levels*”*.*^1^ The specific cut-score of interest in this study is the pass mark, corresponding to the standard of minimal competency to receive a provisional licence to practice. This licence is awarded by the General Medical Council (GMC), which is the official regulatory body for overseeing UK medical education and training. Subsequently, medical graduates have the opportunity to apply for postgraduate training posts to become fully licensed and later, to pursue their medical professions, in locations throughout the UK. This practice carries the implicit assumption that UK medical graduates are of equivalent competence. However, the question remains as to how readily the credibility of this assumption can be tested. A UK study involving use of the Angoff method with the same six-station OSCE for five medical schools detected a marked disparity in pass marks across schools. On this basis, it was argued that a student who would fail this assessment at one medical school would instead pass if they sat the same assessment at another medical school.^2^ A follow-up study demonstrated that use of a Borderline Group (BG) or Borderline Regression (BR) approach to standard setting instead may not eliminate this type of problem.^3^ Boursicot and colleagues^2^^,^^3^ have postulated that such findings may point to different conceptions of minimal competency across medical schools. However, it is difficult to imagine how stable conceptions of minimal competency could be captured for individual schools as a means of testing this hypothesis.

Indeed, it could equally well be argued that the substantial discrepancies in pass marks across schools brings into question the validity of the particular standard setting techniques used. Whichever interpretation of the study findings is the more credible, it is of no less importance to note that the same findings demonstrate a lack of evidence that cut-offs for defining the pass mark across different schools converge towards a common construct – namely, the true standard for defining minimal competence. It is this corollary in particular which adds weight to existing concerns that in the absence of a national licensing exam, there is no benchmark for confirming that UK medical graduates have achieved a common standard of competence on graduation.

It is also of importance to consider the feasibility of comparing standards across schools in real rather than experimental settings based on the types of assessment and standard setting techniques already used by these schools. McCrorie et al. have demonstrated a wide variation between medical schools in the UK in their choice of written and clinical graduating examinations, leading them to “question whether is it possible to make plausible comparisons in relation to the equivalence of standards of graduates from the different UK medical schools”, and to recommend that “national qualifying level examinations should be considered in the UK” as a means of quality assurance.^4^

The case for a national and indeed, a European, licensing exam has been the topic of much debate within medical education communities.^5^^,^^6^ Despite the relative merits of both sides of the debate, there is a need for further work exploring McCrorie et al.’s concern about the feasibility of comparing standards across medical schools. There is therefore a call for a more thorough investigation of the extent of variation in assessment styles across UK medical schools. Furthermore, this ought to be supported by a review of variation in corresponding standard setting methods.

While the GMC carried out a paper-based review of approaches to assessment across 31 UK medical schools in 2013-2014, the content of their follow-up report^7^ is highly anecdotal. Also, there appears to have been variation across schools in the level of transparency with which they reported their assessment strategies, which may explain the absence of data presenting an overview of variation across the schools. However, even where feedback was forthcoming, the GMC report “considerable variation in how medical schools approach assessment.”^7^ In turn, in consistency with the author’s own viewpoint, they raise the concern that “variation can lead to uncertainty as to whether all students are meeting an overall standard”.^7^ This concern invites a more comprehensive overview of differences across schools, including estimates of the prevalence of different assessment practices and encouraging better consistency of input across the participating schools.

The principal aim of this survey-based study, therefore, is to provide an up-to-date synopsis of variation in assessment and standard setting practices across UK medical schools. This is with the intention of providing an indication of the level of transition that would be required in principle to make such practices more uniform across schools and allow standards to be realistically compared. Such work ought to provide a stronger evidence base for evaluating the feasibility of recognizing equity of standards across medical schools in the absence of a national licensing exam. As such, it ought to provide a timely response to the General Medical Council’s recent approval of “a plan to work with partners to develop a unified assessment for every doctor seeking to practise in the UK”. This assessment – the United Kingdom Medical Licensing Assessment (UKMLA) – is to serve as “an international benchmark test for entry to medicine”.^8^ The current study will contribute to the evidence base for assessing the need for such a benchmark.

## Methods

### Literature searching

A PubMed search was conducted using the terms “standard setting” and “medical education” in order to search for newer methods of standard setting within the medical education literature. Bibliographies of relevant journal articles were also consulted, together with the books ‘ABC of Learning and Teaching in Medicine’^9^ and ‘A Practical Guide for Medical Teachers’^10^, with a view to identifying papers highlighting new assessment and standard setting methods.

### Use of terminology

A section entitled ‘Terminology list’ was included early on in the study questionnaire. This list was created with an appreciation of the potential for non-conformity in use of assessment terminology across medical schools and the resultant need to define various terms in advance of their use. For example, Finals were defined as the “final diet of summative exams taken as a requirement for graduation” and the alternative terms "Final Exam", "Final Professional Exam", "Graduating Exam" and "Exit Exam" were listed as conveying the same meaning. Definitions of terms for abbreviating different assessment styles were also provided, together with the list of sub-types of assessment styles over which these terms ranged. The interpretations presented under the above section in the questionnaire are replicated below for ease of reference:

**MCQ:** multiple-choice questions, where examinees are to choose from a list of possible responses. Examples: single best answer questions, extended matching questions, true/false questions.

**SAQ:** (Short Answer Questions): open ended, semi-structured questions, where an examinee's response is expected to be less than 50 words. This concept extends to variants of standard SAQs, including modified essay questions and constructed response questions.

**OSLER:** Objective Structured Long Examination Record, or any of its variants.

**PACES:** Practical Assessment of Clinical Examination Skills, or any exam based on the current MRCP PACES examination.

**OSCE:** Objective Structured Clinical Examination, or any of its variants, except for OSLER or PACES.

**OSPE:** Objective Structured Practical Examination, or any of its variants.

**mini-CEX:** mini Clinical Evaluation Exercise

**DOPS:** Direct Observation of Clinical Practice

**SSC: **Student-Selected Component

In designing the questionnaire, it was also appreciated that there was unlikely to be a clean divide between non-clinical and clinical learning in the transition from early to later years. Therefore, *clinical years* were provisionally defined as pertaining to “the final 3 years of the traditional 5-year medical degree or when students undertake their clinical training”. Correspondingly, *preclinical years* were presented as pertaining to “any year of the medical degree which precedes the Clinical Years”. Respondents were invited to specify any discrepancies between the interpretation offered of clinical years and practices within their own school.

### Recruitment of respondents

This project received the formal approval of the UK Medical Schools Council Assessment Alliance (MSCAA). All recipients of the study questionnaire had agreed in writing to be the contact(s) on behalf of their school prior to commencement of the survey.

The survey respondents were Medical Education professionals with responsibilities for assessment at their respective schools. They were identified through a rigorous process involving contacting the target group of all 34 schools directly and gaining consent from the appropriate assessment specialist to include them or their recommended colleague in the survey contact list. Where contact details for potential survey participants were not explicit from university webpages, advice was sought, initially from ASME (Association for the Study of Medical Education) and later, from the MSCAA, as to the most suitable lines of contact. In all cases, recommended contacts gave consent prior to receipt of a formal survey invitation. The MSCAA also supported the recruitment of respondents through an entry in their October 2013 newsletter.

### Finalizing the study questionnaire

The questionnaire was reviewed by two of the above consenting participants and a measurement theorist, all of whom had previously consented by email to participate in test-runs of the survey. Feedback from this process was productive in enhancing the quality of the study questionnaire in terms of a) clarity of terminology used, b) adequacy of response options for assessment types and standard setting methods and c) signposting to respondents what to expect in later questions of the questionnaire. These criteria were designed to enhance the content validity of the questionnaire response data in relation to capturing a profile of assessment styles and corresponding standard setting methodologies across UK medical schools.

### Overview of questionnaire

An online draft questionnaire was designed using a secure online survey system to include a comprehensive range of assessment styles and standard setting methods. Two-dimensional matrix-style questions, involving standard setting methods along the rows and styles of assessment along the columns, were used to collect response data on summative assessment separately for each of Finals, clinical years (with the exception of finals), pre-clinical years for graduate entry programmes and pre-clinical years for non-graduate programmes. For each of the above stages of assessment, a similar question was used. The stem for Finals is provided here by way of example and for ease of reference:

“Please select ALL the methods your medical school currently uses for setting the pass standard in summative assessments specifically for Finals ***(Later in the survey, you will have the opportunity to respond separately in relation to each of exams which are not Finals in clinical years and exams in nonclinical years)***.”

For completeness, a supplementary question was appended to each of the above matrix questions, with a corresponding free text box. The stem for this supplementary question, which was similar for all of the above stages of assessment, is included for the case of Finals below for convenience:

“Please describe any assessment method used by your medical school in Finals that is not listed above, and the corresponding standard setting method, regardless of whether it is listed above or not”.

Questions inviting free text responses were included in order to obtain feedback, where appropriate, on how standard setting procedures were combined at the various stages of assessment. Further questions explored the weighting of different assessment methods in Finals, use of compensatory and conjunctive approaches “for managing the results of the various components of Finals”, “algorithms used to determine whether or not students graduate” and use of norm-referencing methods.

The later questions, inviting views on national and shared assessment, will be the focus of a separate paper.

### Collection of response data

Further to an initial briefing email, the survey was opened on 13 January 2014, with those who had already agreed to participate being advised to respond by midnight on 23 January 2014. Shortly thereafter, all respondents were encouraged to review their responses for accuracy and completeness and to provide an indication of any extra time required to fully address these issues, with one contact being successfully reminded to respond to the survey. The resultant amendments were completed during the following week.

### Data preparation and presentation of findings

The response data were downloaded from the survey system in csv format and then stored in Ms Excel files for the purposes of presentation and analysis of findings. As assessment practices for clinical years were shared by two of the participating medical schools, in determining response rates for these years, the denominator, or finite population size, was taken to be 32 rather than 33 (the number of UK medical schools offering Finals).

Data corresponding to prevalence of assessment styles and standard setting methods were summarized in table form by means of frequencies and corresponding percentages. As measures of effect size, percentages serve as a useful means of allowing comparisons across groups of varying sample size, which in this study, includes the groups pertaining to different stages of assessment and uptake of different standard setting methods or assessment styles.

For each stage of assessment, there was a fixed target population of potential respondents corresponding to a finite population of modest size (32 or 34), from which a given sample of schools responded to the relevant survey questions. This special case of sampling is distinct from the more traditional case for use of confidence intervals, involving a population for which we have no specific upper bound and which we implicitly treat as infinite. Nevertheless, it is still subject to sampling error and there is a corresponding need to identify an appropriate choice of confidence interval to accurately estimate the standard error for the sample proportion, while noting that in this instance, sampling is without replacement. Further, this study is intended to provide an overview of assessment styles and corresponding standard setting practices across all UK medical schools and not all eligible schools responded for any one stage of assessment. Thus, it is inevitable that, just as with the more traditional case specified above, inferences will require to be made from the respondent samples to the corresponding finite populations. These observations provide the rationale for choice of the Wilson score method^11^ with a correction for finite populations^12^ for calculation of confidence intervals.

### Internal consistency, clarity and completeness

All responses were carefully scrutinized to ensure that respondents had answered the questions as stated and that the intended meaning in their responses was clear. Where responses were open to interpretation, the original respondents were contacted directly to obtain a more accurate representation of assessment practice. This included verifying those rare instances where standard setting methods were used in combination for the same assessment. In a few cases, instances of missing data relating to types of assessment and corresponding standard setting practices were readily addressed through consulting the relevant university webpages. In one instance, this led to the need to consult the Director of Medical Education for the respective school in order to improve the accuracy of the response data originally provided by their colleague, particularly in relation to choice of standard setting methods.

## Results

Of the 34 UK medical schools, 27 (79.4%) agreed to participate. Of the 7 schools which decided not to take part, 2 said they were too busy, while 5 others did not reply to email invitations. Of the 27 participating schools, one school reported that they did not offer Finals.

### Finals

Overall, out of a possible 32 schools, 26 (81.3%) provided responses for questions on Finals. The distribution of assessment styles used for summative assessment in Finals is summarized in Table 1.

**Table 1:** Assessment styles for Finals ordered according to popularity (n = 26)

Assessment style*	Frequency (%)	95% CI**
MCQ	24 (92.3)	(86.5, 95.7)
OSCE	25 (96.2)	(91.4, 98.3)
SAQ	9 (34.6)	(27.1, 43.0)
MiniCeX	3 (11.5)	(7.2, 18.0)
OSLER	3 (11.5)	(7.2, 18.0)
Essay	2 (7.7)	(4.3, 13.5)
Portfolio	2 (7.7)	(4.3, 13.5)
Portfolio viva	2 (7.7)	(4.3, 13.5)
DOPS	1 (3.8)	(1.7, 8.6)
Long case	1 (3.8)	(1.7, 8.6)
Ward simulation exercise†	1 (3.8)	(1.7, 8.6)

*Abbreviations in row headers are defined in the methods section of this paper under 'Use of terminology'. **95% CIs were calculated using the Wilson score method11 with a correction for finite populations.12 †This category was volunteered by a respondent under ‘Other’ and hence added retrospectively to the response options in the presentation of findings.

The distribution of standard setting approaches used according to assessment style for Finals is presented in Table 2.

In Table 2, the column totals for ‘MCQ’ and ‘OSCE’ are of value two greater than the number of schools reporting use of the respective assessment styles (cf. Table 1). This can be explained as follows. Two of the respondent medical schools differentiated between two types of MCQ assessment (e.g. as with the DOSCE and a more traditional MCQ exam). In the case of the OSCE, one school distinguished between their paediatric OSCE and their bespoke form of OSCE which focused on verbal communication with the patient rather than a physical examination.

**Table 2:** Choice of standard setting method(s) according to assessment style for Finals*

Standard setting method	MCQ	OSCE	SAQ	MiniCeX	OSLER	Essay	Portfolio	Portfolio viva	DOPS	Long case	Ward simulation exercise	Total
	Frequency (%)											
Anchor statements with common marking scheme	1 (3.8)	2 (7.4)	-	2 (66.7)	1 (33.3)	1 (50)	1 (50)	-	1 (100)	-	-	9 (11.7)
Anchor statements with common marking scheme and fixed pass mark	-	-	-	1 (33.3)	-	-	-	1 (50)	-	-	-	2 (2.6)
Angoff	15 (57.7)	2 (7.4)	7 (77.8)	-	-	1 (50)	-	-	-	-	-	25 (32.5)
Angoff and Hofstee	2 (7.7)	-	-	-	-	-	-	-	-	-	-	2 (2.6)
Borderline group†	-	9 (33.3)	-	-	-	-	-	1 (50)	-	-	-	10 (13.0)
Borderline group and Hofstee	-	1 (3.7)	-	-	-	-	-	-	-	-	-	1 (1.3)
Borderline regression	-	12 (44.4)	-	-	1 (33.3)	-	-	-	-	1 (100)	-	14 (18.2)
Contrasting-groups	-	-	-	-	-	-	1 (50)	-	-	-	-	1 (1.3)
Ebel	5 (19.2)	1 (3.7)	1 (11.1)	-	-	-	-	-	-	-	-	7 (9.1)
Ebel and Rasch analysis^‡^	1 (3.8)	-	-	-	-	-	-	-	-	-	-	1 (1.3)
Fixed pass mark	1 (3.8)	-	-	-	1 (33.3)	-	-	-	-	-	-	2 (2.6)
Hofstee	1 (3.8)	-	1 (11.1)	-	-	-	-	-	-	-	-	2 (2.6)
Rasch analysis	-	-	-	-	-	-	-	-	-	-	1 (100)	1 (1.3)
Total	26 (100)	27 (100)	9 (100)	3 (100)	3 (100)	2 (100)	2 (100)	2 (100)	1 (100)	1 (100)	1 (100)	77 (100)

*Abbreviations in column headers are defined in the methods section of this paper under 'Use of terminology'. The symbol ‘-’ is used to denote all instances where the corresponding combination of standard setting technique and assessment style was not selected by any respondent as pertaining to Finals at their medical school. †In conjunction with the OSCE, one school specified use of the median (rather than the mean) in deriving the pass mark. ‡Here, Rasch analysis was presented by the respondent school as being used to monitor or moderate marks derived by the Ebel method.

A further school alluded to variation in choice of standard setting method for their OSCE (either Anchor statements with a common marking scheme alone or an Angoff approach).

Only four schools provided complete information on weightings used for Finals. These weightings, which varied across individual medical schools, were as follows:

   •  33.3% Clinical Practice Examination (hybrid of MiniCeX and traditional OSCE), 33.3% Portfolio Viva, 33.3% Safety in Prescribing and Practice (MCQ)

   •  80% OSCE, 20% mini-CEX

   •  55% OSCE, 45% SAQ

   •  50% MCQ, 50% OSCE

Out of a possible 32 schools, 24 (75%) also provided information on the use of a conjunctive or compensatory approach in managing the various components of Finals, with the conjunctive approach being accepted by all respondents. The tendency here was to view Finals as a single assessment comprised of a range of assessments styles and to indicate that while on the one hand, compensation could not be accommodated across these assessment styles, some degree of compensation occurred within the assessment pertaining to a given style.  Thus, for example, with reference to the OSLER, one school reported that, *“*Within the clinical exam, it is possible to perform poorly on a particular patient case and still pass”. Another school reported that while the MCQ and OSCE assessments included in Finals required to be passed separately, there was no requirement within these exams for specific items to be passed. However, specifications varied as to how a pass was defined. For example, for the OSCE, three schools highlighted similar requirements in terms of their chosen minimum percentage or fraction of stations to be passed (approximately 70%, two-thirds or 11/16). By contrast, another school demonstrated its own unique application of a conjunctive approach in that for their three-part Finals, each part required to be passed separately and that for each of the different assessment styles within each part, a separate pass was required. In addition, a wide range of requirements was reported across medical schools in relation to course assignments and prior assessments in the final and penultimate year that students were expected to pass in order to be permitted to graduate.

### Non-Finals in clinical years

Overall, out of a possible 32 schools, 25 (78.1%) provided responses for questions on non-Final assessments in the clinical years of their medical programme. The distribution of assessments styles used for summative assessment in clinical non-Final assessments is summarized in Table 3. The distribution of standard setting approaches used according to assessment style for clinical non-Finals is presented in Table 4.

### Preclinical years

Overall, 11 out of a possible 16 respondents provided responses for questions on pre-clinical years of (accelerated) Graduate Entry Programmes (GEPs), while 21 out of a possible 32 respondents provided responses for preclinical years of non-graduate entry programmes.

The decision was made to merge the response data for pre-clinical years across graduate and non-graduate entry programmes so as to reflect assessment and standard setting choices for pre-clinical years more generally. This was for two main reasons. Firstly, schools which were eligible to respond for both of these categories were incomplete in their response behaviours, with the potential that this would lead to drawing unjustified conclusions about distinctions in assessment and standard setting practices across these two categories. Secondly, it was reported by a few respondents that there was no difference in such practices across the two categories. Therefore, there was a lack of evidence that separating the two categories was reliable for reporting purposes. It was noted, therefore, that out of a possible 34 schools, 22 (64.7%) provided responses for pre-clinical assessment styles and corresponding standard setting methods. Proportions and corresponding confidence intervals are reported accordingly in Table 3.

**Table 3:** Assessment styles for non-Finals in clinical years and for pre-clinical years

Assessment style*	Non-Finals in clinical years (n=25)		Pre-clinical years (n=22)	
	Frequency (%)	95% CI**	Frequency (%)	95%CI**
MCQ	25 (100)	(96.6, 100)	22 (100)	(94.0, 100)
OSCE	25 (100)	(96.6, 100)	21 (95.5)	(87.0, 98.5)
SSC	18 (72.0)	(63.0, 79.5)	13 (59.1)	(46.5, 70.6)
SAQ	15 (60.0)	(50.7, 68.6)	15 (68.2)	(55.7, 78.5)
Portfolio	8 (32.0)	(24.0, 41.2)	5 (22.7)	(14.0, 34.7)
Essay	6 (24.0)	(17.0, 32.7)	8 (36.4)	(25.4, 49.0)
OSPE/Anatomy spot exam	6 (24.0)	(17.0, 32.7)	8 (36.4)	(25.4, 49.0)
MiniCeX	5 (20.0)	(13.6, 28.4)	1 (4.5)	(1.5, 13.0)
Oral presentations	5 (20.0)	(13.6, 28.4)	2 (9.1)	(4.1, 19.0)
In vitro clinical competencies^†^	2 (8.0)	(4.2, 14.6)	1 (4.5)	(1.5, 13.0)
OSLER	2 (8.0)	(4.2, 14.6)	0 (0.0)	(0, 6.0)
Viva (other)	1 (4.0)	(1.6, 9.4)	0 (0.0)	(0, 6.0)

*Abbreviations in row headers are defined in the methods section of this paper under 'Use of terminology'. **95% CIs were calculated using the Wilson score method11 with a correction for finite populations12. †This category was volunteered by a respondent under ‘Other’ and hence added retrospectively to the response options in the presentation of findings.

The distribution of standard setting approaches according to assessment style for pre-clinical years is presented in Table 5.

### Norm referencing

Twnenty six out of a possible 34 (76.5%) respondents responded to questions about norm referencing. Of these, 6 respondents (23.1%; 95% CI: 16.1%, 31.9%) indicated they used norm-referencing methods in their medical schools. For two of these schools, this included use of norm referencing for progress tests only. For another two schools, respondents specified that norm referencing was used to create a “borderline” group of students. Here, one school used this approach to specify the requirement, exclusively for Years 1 - 2, that one borderline pass was allowable in order to progress. By contrast, the other school defined a borderline group for all stages of assessment. In the latter two cases, a borderline pass was defined relative to the original pass mark derived through one of the standard setting methods listed in the survey matrices, with the first (second) school defining the borderline group as falling 1 SEM (1- 2 SEM, respectively) below the original pass mark.

Four respondents who recorded that their school did not use norm referencing at any stage of summative assessment took the opportunity to express their viewpoint that this method was inappropriate and indeed, in two such cases, that its use was against University policy. Dissenters also appealed to “a wide body of literature”, “graduating competent students” or “best practice” or adopted a tutor-like tone according to which criterion referenced assessment rather than norm referencing was deemed to be the choice of the learned in Medical Education assessment. By contrast, a respondent whose school used norm referencing exclusively for progress testing noted that, “Our method avoids the standard objection to norm-referencing, which is that students must fail: in our system, if a student follows an unsatisfactory mark with any better mark, the student progresses.”

### Further sources of variation evident from free text responses

It was implicit from text-based responses that extent of usage and the range of rules of application for sequential and progress testing and the corresponding subject specialisms for which they were used was likely to have varied considerably across respondent schools.

In the case of Finals, one school explained that for their OSLER, they hoped to move to using the BR method in lieu of a fixed pass mark (which they defined as a threshold based on “historic evidence”). This same school reported use of a penalty points marking scheme with the OSLER, both for Finals and non-Finals. One school also reported the practice of carrying over a failed OSCE assessment for re-sitting in the final year, while another school reported choice of the penultimate rather than the final year as the year for sitting Finals. In the latter case, the same school required students to successfully complete portfolio assignments in their final year, rather than, as for the majority of other schools, in advance of Finals.

**Table 4:** Choice of standard setting method(s) according to assessment style for clinical non-Finals*

Standard setting method	MCQ	OSCE	SAQ	MiniCeX	OSLER	Essay	Portfolio	DOPS	Long case	OSPE/Anatomyspot exam	Oral presentations	SSC	Viva(Other)	Other	Total
	Frequency (%)^†^														
Anchor statements with common marking scheme (CMS)	1 (4.5)	-	2 (13.3)	5 (100)	-	2 (33.3)	7 (87.5)	1 (100)	1 (100)	1 (20)	5 (100)	11 (64.7)	1 (100)	2 (40)	39 (32.5)
Anchor statements with CMS and Fixed pass mark	1 (4.5)	2 (7.4)	-	-	-	1 (16.7)	-	-	-	-	-	3 (17.6)	-	2 (40)	9 (7.5)
Angoff	16 (72.7)	3 (11.1)	12 (80)	-	-	1 (16.7)	-	-	-	3 (60)	-	1 (5.9)	-	-	36 (30)
Borderline group	-	7 (25.9)	-	-	-	-	-	-	-	-	-	-	-	-	7 (5.8)
Borderline group and Hofstee^‡^	-	1 (3.7)	-	-	-	-	-	-	-	-	-	-	-	-	1 (0.8)
Borderline regression	1 (4.5)	14 (51.9)	-	-	1 (50)	-	-	-	-	-	-	1 (5.9)	-	-	17 (14.2)
Contrasting-groups	-	-	-	-	-	-	1 (12.5)	-	-	-	-	-	-	-	1 (0.8)
Ebel	-	-	-	-	-	-	-	-	-	1 (20)	-	-	-	-	1 (0.8)
Fixed pass mark	1 (4.5)	-	-	-	1 (50)	2 (33.3)	-	-	-	-	-	1 (5.9)	-	-	5 (4.2)
Hofstee	2 (9.1)	-	1 (6.7)	-	-	-	-	-	-	-	-	-	-	1 (20)	4 (3.3)
Total	22 (100)	27 (100)	15 (100)	5 (100)	2 (100)	6 (100)	8 (100)	1 (100)	1 (100)	5 (100)	5 (100)	17 (100)	1 (100)	5 (100)	120

*Abbreviations in column headers are defined in the methods section of this paper under 'Use of terminology'. The symbol ‘-’ is used to denote all instances where the corresponding combination of standard setting technique and assessment style was not selected by any respondent as pertaining to clinical non-Finals at their medical school. †Frequencies and percentages pertain to instances of use of the given standard setting approach for the listed assessment type and as such, may include multiple instances for a given medical school. ‡Here, it was reported that the two standard setting methods were applied separately for the first of two phases of the assessment and the higher of the resultant two pass marks assigned to this first phase of the assessment. Interestingly, students who did not pass this first phase of assessment would in turn require to sit a second phase for which the pass mark was determined using an Angoff approach.

**Table 5:** Choice of standard setting method(s) according to assessment style for pre-clinical years*

Standard setting method	MCQ	OSCE	SAQ	MiniCeX	OSLER	Essay	Portfolio	Portfolio viva	DOPS	Long case	Ward simulation exercise	OSPE/ Anatomy spot exam	SSC	Total
	Frequency (%)^†^													
Anchor Statements with common marking scheme (CMS)	1 (3.3)	2 (6.9)	1 (7.7)	2 (66.7)	1 (33.3)	1 (20)	1 (100)	-	1 (100)	-	-	2 (40)	1 (100)	13 (13.7)
Anchor statements with CMS and fixed pass mark	-	-	-	1 (33.3)	-	-	-	1 (50)	-	-	-	-	-	2 (2.1)
Angoff	16 (53.3)	2 (6.9)	8 (61.5)	-	-	1 (20)	-	-	-	-	-	1 (20)	-	28 (29.5)
Angoff and Hofstee	2 (6.7)	-	-	-	-	-	-	-	-	-	-	-	-	2 (2.1)
Borderline group	-	11 (37.9)	-	-	-	-	-	1 (50)	-	-	-	-	-	12 (12.6)
Borderline group and Hofstee	-	1 (3.4)	-	-	-	-	-	-	-	-	-	-	-	1 (1.1)
Borderline regression	-	12 (41.4)	-	-	1 (33.3)	-	-	-	-	1 (100)	-	-	-	14 (14.7)
Ebel	5 (16.7)	1 (3.4)	1 (7.7)	-	-	-	-	-	-	-	-	-	-	7 (7.4)
Ebel and Rasch analysis	1 (3.3)	-	-	-	-	-	-	-	-	-	-	-	-	1 (1.1)
Fixed pass mark	3 (10.0)	-	2 (15.4)	-	1 (33.3)	3 (60)	-	-	-	-	-	-	-	9 (9.5)
Hofstee	2 (6.7)	-	1 (7.7)	-	-	-	-	-	-	-	-	2 (40)	-	5 (5.3)
Rasch analysis	-	-	-	-	-	-	-	-	-	-	1 (100)	-	-	1 (1.1)
Total	30 (100)	29 (100)	13 (100)	3 (100)	3 (100)	5 (100)	1 (100)	2 (100)	1 (100)	1 (100)	1 (100)	5 (100)	1 (100)	95 (100)

*Abbreviations in column headers are defined in the methods section of this paper under 'Use of terminology'. The symbol ‘-’ is used to denote all instances where the corresponding combination of standard setting technique and assessment style was not selected by any respondent as pertaining to pre-clinical years at their medical school. †Frequencies and percentages pertain to instances of use of the given standard setting approach for the listed assessment type and as such, may include multiple instances for a given medical school.

One school also indicated the intention of introducing the Hofstee method in the next academic year for use with clinical non-Finals. Likewise, another school indicated the intention of introducing a new MCQ exam into Finals in the coming year and of using a combined Angoff-Hofstee approach rather than adopting their traditional choice for this assessment style of using an Angoff approach only. A further school reported that in the subsequent academic year, it was their intention to use the Cohen method for progress tests in Years 2 - 4.

## Discussion

### Assessment styles

MCQs and SAQs were the most commonly found written assessments, used widely from preclinical years through to Finals (Tables 1 and 3), with Student Selected Components (SSCs), in their various forms, proving particularly popular for non-Final assessment in clinical years (Table 3).  While the choice of assessment styles for matrices used within the questionnaire was informed both by the medical education literature and through test-running the survey, it was clear from individual text responses that even for a given assessment style, a school could be distinctive in its delivery. For example, the lesser known assessment style DOSCE (Data Objective Structured Clinical Examination) – “a picture/recording based clinical assessment with a one best answer sheet” fell under the universal type ‘MCQ’. Further, the original OSCE has been subject to many innovations over the last decade, leading in many cases, to schools having their own special variants, which are arguably becoming less easy to classify under a standard assessment type. One of several such cases for the response data was reported for Finals. In this particular instance, the assessment appeared, more precisely, to take the form of a hybrid assessment. This assessment drew from the strengths of the MiniCeX through, for example, use of physical examinations involving authentic real patient cases and the requirement to provide a diagnosis and treatment management plan. However, it also drew from the strengths of the traditional OSCE through, for example, inclusion of a structured approach involving assessment over a broad range of course elements.

While the above illustration of innovation in the implementation of assessment styles may provide an effective compromise in responding to Norcini’s original recommendation that “[t]he MiniCeX is not intended for use in a high-stakes examination setting”,^13^ it is noteworthy that the MiniCeX was also reported explicitly as a choice for Finals for three schools (Table 1). Thus, it would appear that differences in assessment styles across schools are rooted, not only in resource constraints, but also, in the philosophical convictions of medical educators concerning the rationale for choosing one assessment style in lieu of another.

Variation in implementation of specific assessment styles was also illustrated for the case of SSCs, with one school reporting that students embark on SSCs through joining honours programmes. The sustainability of SSCs as a requirement for progression to Finals might also need to be questioned, given that, for example, one school reported the intention of removing SSCs from their programme in the next academic year. Additionally, the challenge of having SAQs marked in a “timely” manner led one school to report their intention of eventually eliminating this assessment style from assessment for Finals.

### Standard setting methods

A number of observations regarding usage of standard setting methods are forthcoming from Tables 2, 4 and 5, both in terms of popularity and diversity across schools. At any one stage of assessment, at least 60% of all MCQs and SAQs were reported as having been standard set using an Angoff approach. Anchor statements with a common marking scheme, either with or without a fixed pass mark, proved popular with less common assessment styles, but also, with SSCs.

While use of SSCs and portfolios was evident at all stages of assessment, they featured mainly as part of summative assessment in the clinical years. Most portfolio and SSC assessments were standard set using anchor statements with a common marking scheme; correspondingly this standard setting method proved the more popular overall for clinical non-Finals.

Standard setting for the OSCE was limited to one of three types - Angoff, BG, and BR methods, with most schools using either of the latter two methods. While the borderline methods appeared to be almost equally popular with OSCEs, the BR method was notably the more popular for this assessment style in later years, particularly in the case of clinical non-Finals, where the BR method appeared just over twice as often overall as the BG method (Table 4).

By contrast, for MCQs (Tables 2, 4 and 5), Angoff approaches (including combined Angoff and Hofstee approaches) proved particularly popular, with usage ranging from 60% of cases (pre-clincal assessments) to almost 73% of cases (clinical non-Finals). Even with clinical non-Finals, however, where the corresponding percentage uptake was relatively high, the choice was by no means unanimous and for the remaining six schools (27.3% of cases), preferred choices for MCQs ranged over five alternative standard setting approaches (Table 4).

Interestingly, combined standard setting methods, involving either the Hofstee method or Rasch analysis were used exclusively with MCQs and OSCEs, with the intention being to moderate or monitor pass-marks based on Angoff, BG or Ebel techniques.

Of the above three methods, the Ebel method is unique in presenting standard setters with the task of making judgment on a test item’s relevance and of separately but simultaneously making a judgement on the item’s difficulty. Arguably, the task of separating out the corresponding standard setting tasks is a challenging one.^14^ Nevertheless, the current standard setting data (Tables 2, 4 and 5) indicate that the Ebel method remains in use in medical education assessment today. Indeed, in two cases, effort has been made to combine this particular method with established techniques, rather than supersede it with alternative choices (of which there are many). These two cases pertain to the same medical school and the respondent for this particular school stressed that they did not wish to distinguish between non-clinical and clinical years according to content. Nevertheless, as the respondent did not offer an alternative definition for clinical years to that provided in the questionnaire, it is necessary to assume that these instances pertain both to assessment for the first two years of the degree programme for undergraduate entrants and to Finals. These findings suggest that for a small subset of the respondent medical schools, this particular standard setting method has earned the respect of faculty members and that for this reason, they are unlikely to wish to dispense with it in the immediate term. Furthermore, the low usage or non-uptake of other standard setting methods, such as the Contrasting-Groups and Cohen methods, need not be a reflection of their future demise, as is illustrated by one school’s intention for future uptake of Cohen’s method.

Schools varied, not only according to choice of standard setting method, however, but also, according to how these methods were implemented for a given assessment style. For some assessment styles, such as the OSCE, separate components were identified and standard set using distinct standard setting techniques, with the resultant pass marks being combined to form an overall pass mark. Such practice was not consistent across schools and ought to be distinguished from that of combining two standard setting techniques to form a single pass mark. (It is the latter procedure which is intended in Tables 2, 4 and 5 wherever two standard setting methods are listed jointly in the same row, such as for the entry ‘Angoff and Hofstee’ of Table 5.)

Further, while the Angoff method has proliferated in its various subtypes over many years, the Borderline methods are also open to variation, as is illustrated by one respondent’s choice to highlight usage of the median rather than the mean in determining the pass mark with the BG method (Table 2).

### Further sources of variation

Intended changes in assessment and standard setting practices of varying types have also been reported, as have school initiatives in choice of algorithms for deriving test scores, an example of which is the use of penalty points. It is unclear from the response data that use of penalty points would be the preferred choice for all schools in the allocation of individual test scores for any one assessment style or at any one stage of assessment. The carrying over of a failed OSCE assessment for re-sitting in final year is a further example of an assessment strategy which may be viewed as leading to inconsistency in standards for progression to Finals. Inconsistency was also evident across schools regarding the choice of year for Finals – final versus penultimate year.

### Norm referencing

Of those medical schools which responded to the questions on norm-referencing, just less than a quarter indicated that they used norm-referencing methods. Arguably, the unpopularity of norm referencing in medical education rests fundamentally on the principle that medical education is competency based. The decision as to whether competency has been achieved for an individual examinee should not rest on the fluctuating abilities of examinee cohorts but rather, on the performance of that individual examinee relative to a stable cut-off which is grounded on test content. Nevertheless, on a few occasions specific contexts were highlighted for its use. These included progress testing (see earlier), with one respondent highlighting their justification for opposing an alternative perspective on norm-referencing. A further context was that of defining a boundary for the original pass mark, which lends support to the following viewpoint expressed in the medical education literature: “A compromise method, combining a pre-fixed cut-off score with a relative point of reference, reduces the disadvantages of conventional criterion and norm-referenced methods, whilst making optimal use of the advantages”.^15^

### Compensatory and conjunctive approaches and weighting for Finals

Views on the usage of compensatory versus conjunctive approaches for Finals were fairly uniform. Compensatory approaches across individual assessments within Finals did not feature within the response data and such approaches were mainly applied within the individual assessments, thus avoiding “sudden death” questions. Nevertheless, through defining Finals as an assessment in parts, one school had applied a conjunctive approach, not only to the assessment parts but also, to the separate assessment styles within these parts. This particular case reflects the level of uniqueness in approaches to assessing undergraduate medical students that can evolve where schools are at liberty to develop their own brands of assessment.

Data on the weighting of assessment components for Finals were notably lacking by comparison with other response data, most likely due to difficulties in accessing this more accurate information. However, even for the four participating schools, there was evidence that, for this particular aspect of assessment, views were divided. For example, one school attributed considerable weight to SAQs in Finals, while another school expressed the intention to phase out SAQ assessments from summative assessment in clinical years on account of the associated workload for assessors.

### Limitations

Seven of the eligible UK medical schools failed to participate in the study, so while, given the competing interests faced by Medical Education professionals, the response rate was surprisingly high, the survey findings may not be fully representative of medical education assessment practices in the UK.

It was recognized from the outset that the subdivision of medical programmes into clinical and preclinical stages was likely to be an over-simplification in many cases. However, this was addressed early on in the questionnaire through allowing respondents to add appropriate content to represent their respective schools more accurately. Those who did respond to this opportunity (12 respondents) mainly highlighted programmes involving full or partial integration of clinical teaching with non-clinical teaching in early years (11 respondents). However, the questionnaire matrices accommodated such cases by offering the same standard setting methods and assessment styles to candidates irrespective of the stage of assessment. Further, where only accelerated graduate entry programmes were on offer, more clinically focused learning would have required to have occurred sooner for their graduate entrants than in the case of a traditional 5-year undergraduate programme. Nevertheless, this should not have inhibited provision of separate responses for pre-clinical and clinical years for such programmes.

Based on the widespread recognition of the utility of the Rasch model in the medical education literature,^16^-^18^ it is also possible that while Rasch analysis was rarely aligned with a particular assessment style via the response matrices, it was still recognized as a useful means of evaluating the performance of an established standard setting method within a given school. For example, one respondent chose to distinguish between their “mainstream” standard setting method and the use of Rasch analysis “to look at equating/anchor/how well our estimates fit with ability over time.” Such practice at other schools may not have been fully captured by the response data.

### Future work

The survey reported on in this paper was also used to glean the views of respondents on shared assessment and national licensing examinations. The findings from this particular aspect of the study will form the basis for a subsequent paper involving the statistical analysis of levels of agreement across schools (using Likert scale data) and the examination of respondents’ perspectives on each of these areas.

Also, in their 2014 report,^8^ the GMC record having observed variation in approaches to assessment across UK medical schools according to factors additional to standard setting practices and assessment styles. Examples include “timing of final year assessments”, commitment to use of blueprinting to map intended learning outcomes in exams to GMC “competence domains”, training of staff in assessment practices, clarity of policies for student progression through programmes (including “number of resits allowed and the time a student can take to complete their programme”) and amount of teaching and assessment on professionalism. Arguably, variation in any of these factors has the potential to undermine achievement of a common standard and there is a corresponding call to make the extent of variation across schools more transparent and accessible to stakeholders.

## Conclusions

This study demonstrated quite obvious trends of usage regarding assessment methods and standard setting methods. For written assessments, MCQs and SAQs were the most commonly employed formats. They were mostly standard set using the Angoff method, although some schools used the Ebel and Hofstee methods in early or later years. OSCEs were the most commonly used clinical assessment method, and were normally standard set using the BG or BR method. SSCs and portfolios were mostly standard set with anchor statements and a common marking scheme.

However, more generally, there was a marked disparity in assessment and standard setting practices across UK undergraduate medical schools. Additional sources of variation in assessment practices were evident from the response data. In particular, while use of a conjunctive approach in deciding the pass conditions for Finals was the norm, the underlying variation in the structure defining how assessment styles and parts were nested into Finals indicates that there was inconsistency across schools in the choice of algorithm for deriving examinee scores for Finals. Inconsistency was also evident across schools through the emergence of hybrid standard setting methods and bespoke practices involving use of elements of different assessment styles within a single examination and use of different types of standard setting method for different components of the same OSCE. The range of perspectives represented by those who did and did not use norm-referencing for summative assessment was a further source of variation across schools.

These findings highlight the difficulty that prevails in obtaining reassurance that on face value, UK medical schools adhere to a common standard of minimal competency for their medical graduates. In so far as the introduction of a national licensing exam could help in alleviating this difficulty, this study lends support to the GMC’s recent approval of the development of a UKMLA.^8^ As this assessment is to be used “for every doctor seeking to practise in the UK”, the GMC’s decision is of international significance. The findings from this study should also invoke an interest among non-UK medical educationalists to explore the likely variation in assessment styles and standard practices across medical schools within their own countries and between countries, thus contributing to future discussions concerning the role of a European licensing exam.

### Acknowledgements

The author is grateful to the two Medical Education specialists from participating medical schools and Professor Gregory Stone (measurement theorist, University of Toledo) for their time and input in reviewing a draft of the study questionnaire. Thanks are also due to Professor Helen Cameron, Ms Marshall Dozier (University of Edinburgh) and Dr Ali Alnoor Kara (University of Western Ontario) who advised on the design of an earlier version of the study questionnaire, Dr Lim Ming Han (University of Edinburgh) who contributed to questionnaire design and the identification of relevant background literature for this study, Professor Robert G Newcombe (Cardiff University) for a useful discussion on suitable methodology for calculating confidence intervals and to the following persons for their commitment in identifying suitable respondents for the survey: Ms Nicky Pender (Association for the Study of Medical Education), Ms Veronica Davids (Medical Schools Council Assessment Alliance),who also assisted in promoting the survey, and colleagues of survey respondents within the respective medical schools. The author extends their thanks to the journal reviewers for their feedback, which has assisted greatly in enhancing the overall quality, and in particular, the clarity of content of this paper.

### Conflict of Interest

The author declares that they have no conflict of interest.
